# *NPRL2* Sensitizes Human Non-Small Cell Lung Cancer (NSCLC) Cells to Cisplatin Treatment by Regulating Key Components in the DNA Repair Pathway

**DOI:** 10.1371/journal.pone.0011994

**Published:** 2010-08-05

**Authors:** Gitanjali Jayachandran, Kentaro Ueda, Bingbing Wang, Jack A. Roth, Lin Ji

**Affiliations:** 1 Department of Thoracic and Cardiovascular Surgery, The University of Texas M. D. Anderson Cancer Center, Houston, Texas, United States of America; 2 The University of Texas Graduate School of Biomedical Sciences at Houston, Houston, Texas, United States of America; 3 School of Medicine, Wakayama Medical University, Wakayama, Japan; University Medical Center Hamburg-Eppendorf, Germany

## Abstract

NPRL2, one of the tumor suppressor genes residing in a 120-kb homozygous deletion region of human chromosome band 3p21.3, has a high degree of amino acid sequence homology with the nitrogen permease regulator 2 (NPR2) yeast gene, and mutations of *NPRL2* in yeast cells are associated with resistance to cisplatin-mediated cell killing. Previously, we showed that restoration of NPRL2 in NPRL2-negative and cisplatin-resistant cells resensitize lung cancer cells to cisplatin treatment *in vitro and in vivo*. In this study, we show that sensitization of non-small cell lung cancer (NSCLC) cells to cisplatin by NPRL2 is accomplished through the regulation of key components in the DNA-damage checkpoint pathway. NPRL2 can phosphorylate ataxia telangiectasia mutated (ATM) kinase activated by cisplatin and promote downstream γ-H2AX formation *in vitro* and *in vivo*, which occurs during apoptosis concurrently with the initial appearance of high-molecular-weight DNA fragments. Moreover, this combination treatment results in higher Chk1 and Chk2 kinase activity than does treatment with cisplatin alone and can activate Chk2 in pleural metastases tumor xenograft in mice. Activated Chk1 and Chk2 increase the expression of cell cycle checkpoint proteins, including Cdc25A and Cdc25C, leading to higher levels of G2/M arrest in tumor cells treated with NPRL2 and cisplatin than in tumor cells treated with cisplatin only. Our results therefore suggest that ectopic expression of NPRL2 activates the DNA damage checkpoint pathway in cisplatin-resistant and NPRL2-negative cells; hence, the combination of NPRL2 and cisplatin can resensitize cisplatin nonresponders to cisplatin treatment through the activation of the DNA damage checkpoint pathway, leading to cell arrest in the G2/M phase and induction of apoptosis. The direct implication of this study is that combination treatment with NPRL2 and cisplatin may overcome cisplatin resistance and enhance therapeutic efficacy.

## Introduction

*NPRL2/Gene 21* (GenBank accession #AF040707), which is 1351 bp long and encodes a protein of 380 amino acid residues, is one of the tumor suppressor genes identified in a 120-kb homozygous deletion region on human chromosome band 3p21.3 [1], [2]. The frequent and early loss of heterozygosity and the overlapping homozygous deletions observed in the 3p21.3 region in lung and breast cancers suggest a critical role of one or more 3p21.3 genes in the molecular pathogenesis of these cancers [1], [3].

The nitrogen permease regulator 2 (NPR2) yeast gene (GenBank accession #P39923) was identified as a novel component involved in cell killing triggered by cisplatin. Because disruption of NPR2 was shown to confer resistance to cisplatin, it was believed that NPRL2 may use a similar mechanism to mediate the cytotoxicity of anticancer drugs [4]. We recently found that the reexpression of NPRL2 in NPRL2-negative and cisplatin-resistant cells significantly resensitized the response of these cells to cisplatin treatment, as evidenced by reduced cell viability and increased apoptosis *in vitro* and *in vivo* [5]. However, the molecular events responsible for resensitization to cisplatin by *NPRL2* have not been identified. In this study, we attempt to understand the molecular link between NPRL2 and cisplatin in overcoming drug resistance.

The effects of cisplatin are mediated through high levels of DNA damage, leading to programmed cell death or cell cycle arrest [6]. The double-strand DNA breaks induced by cisplatin are mediated through a central DNA damage-signaling pathway controlled by the ataxia telangiectasia mutated (ATM) kinase as well as several other DNA damage-responsive kinases [7], [8]. The phosphorylation of ATM at serine-1981 has been shown to phosphorylate histone H2AX [9], and the phosphorylation of histone H2AX at serine-139 (γ-H2AX) is essential for the recruitment of mediators such as MDC1 (mediator of DNA damage checkpoint protein 1), 53BP1 (p53 binding protein 1), BRCA1 (breast cancer 1), and MRE11 (meiotic recombination 11)-RAD50 (radiation sensitive 50)-NBS1 (Nijmegen breakage syndrome 1) complex [10]–[13]. Phospho-ATM facilitates the phosphorylation of those mediators, and the formation of nuclear foci of the activated molecules promotes transmission of the DNA damage signal to downstream targets, such as Chk1 (check point kinase 1), Chk2 (check point kinase 2), and SMC1 (structural maintenance of chromosomes 1) [14]–[16]. Chk1 and Chk2 are involved in various DNA-damage responses, including cell-cycle checkpoint, genome maintenance, DNA repair, and apoptosis [17].

Therefore, we hypothesized that the sensitization of non-small cell lung cancer (NSCLC) cells to cisplatin by NPRL2 may be due to the direct regulation of key components in the DNA-damage checkpoint pathway. To test this hypothesis, we analyzed effects of the ectopic expression of NPRL2 in the presence or absence of the DNA-damaging agent cisplatin on tumor cell growth and apoptosis in a panel of NSCLC cell lines with varied status of the endogenous NPRL2 gene and protein expression and cisplatin sensitivity. We show that reexpression of NPRL2 in NPRL2-negative and cisplatin-resistant cells significantly activates those key components, including ATM, Chk, and H2AX, and resensitizes lung cancer cells to cisplatin treatment *in vitro* and *in vivo*, leading to cell cycle arrest in the G2/M phase and induction of apoptosis. The findings of the present study also lend experimental support to our hypothesis that NPRL2 may function not only as a prognostic biomarker for sensitivity to cisplatin, but also as a therapeutic agent for resensitizing nonresponding cancer cells to cisplatin.

## Materials and Methods

### Cell lines and cell culture

The human NSCLC cell lines H1299 [5] and H322 [5], with various 3p21.3 status described previously [18], [19], were used for *in vitro* and *in vivo* experiments. H1299 and H322 are cisplatin-resistant (50% inhibitory concentration [IC_50_] and IC_20_ values for cisplatin in H1299 were 7.6 and 3.0 µM, respectively, and in H322 were 13.1 and 5.0 µM, respectively, as assessed by XTT assay) and do not express NPRL2 protein (as assessed by Western blotting) [5]. The cisplatin-sensitive NSCLC cell line H1437 and cisplatin-resistant cell line H1437R have been described previously [5].

### Antibodies, reagents, and dosage

Anti-ATM (5C2), anti-Chk1 (G-4), anti-Chk2 (H-300), anti-Cdc25A (F-6), anti-cyclin B1 (GNS1), and anti-caspase-2 (H-19) were purchased from Santa Cruz Biotechnology (Santa Cruz, CA). Anti-phospho-Chk1 (Ser345), anti-phospho-Chk2 (Thr68), anti-phospho-Chk1 (Ser345), anti-NBS1, anti-phospho-NBS1 (Ser343), anti-SMC1, anti-phospho-Cdc25C (Ser216), anti-phospho-Cdc2 (Tyr15), anti-caspase-3, and anti-caspase-9 were purchased from Cell Signaling Technology (Beverly, MA). Anti-ATM Protein Kinase pS1981 was from Rockland Immunochemicals (Gilbertsville, PA), anti-phospho-histone H2AX (Ser139) was from Upstate Biotechnology (Lake Placid, NY), anti-phospho-SMC1 was from Novus Biologicals (Littleton, CO), and anti-caspase-8 and anti-poly (ADP-ribose) polymerase (PARP) were from BD Biosciences Pharmingen (San Diego, CA).

For all Western blotting, anti-β-actin (Sigma, St. Louis, MO) was used as loading control. Cisplatin was purchased from Bristol-Myers Squibb Company (Princeton, NJ). DOTAP was used to transfect the plasmid vectors to H1299 and H322 cells. Cisplatin treatment was always administered to cells at the IC_20_ dosage (5). DOTAP:cholesterol (DOTAP:Chol) (20 mM) in 5% dextrose in water, used for *in vivo* experiments, was synthesized by our laboratory with use of standard procedures previously described [5].

### Construction of plasmid vectors expressing NPRL2

NPRL2 cDNA was excised from the plasmid pAd-RAP-NPRL2 [2] containing human NPRL2 cDNA and was ligated into the multicloning site of pdCpG-KGB2 expression vector. The cloning strategy has been previously described [5]. Empty vector (pdCpG-KGB2) and a plasmid vector expressing the LacZ gene were used as negative controls. We had previously confirmed that the transfection by this NPRL2 vector could indeed express exogenous NPRL2 protein *in vitro* and *in vivo* [5] by using anti-NPRL2 antibody purchased from Abcam (Cambridge, MA).

### Immunofluorescence staining and flow cytometric analyses for γ-H2AX

Immunofluorescence staining was performed in cells cultured in chamber slides. At designated time points, H1299 cells treated with NPRL2 with or without cisplatin were fixed in 10% formalin and incubated with anti-phospho-histone H2AX (Ser139) (1∶100) antibodies in 5% phosphate-buffered saline (PBS) with bovine serum albumin for 1 h at room temperature. Subsequently, the cells were incubated with donkey anti-mouse IgG-fluorescein isothiocyanate (FITC) (Santa Cruz Biotechnology) diluted 1∶100 in PBS for 45 min, and the samples were mounted with VECTASHIELD mounting medium with 4′,6-diamidino-2-phenylindole (DAPI) (Vector Laboratories Inc., Burlingame, CA) to counterstain the nuclei. Immediately, the slides were examined with use of a fluorescence microscope. Also, after incubation of the FITC antibody, the cells were harvested, and FITC-positive cells were counted by flow cytometric analysis.

### Chk1 and Chk2 kinase activity assay

Chk1 and Chk2 kinase activity in H1299 cells treated with NPRL2 with or without an IC_20_ value of cisplatin were assessed with use of a K-LISA Checkpoint Activity Kit (EMD Biosciences, San Diego, CA), which is an enzyme-linked immunosorbent assay (ELISA)-based activity assay. Briefly, H1299 cells were plated in 60-mm dishes at 4×10^5^ cells per dish and the following day were treated with 4 µg of empty vector or NPRL2 with or without 3.0 µM cisplatin. After 72 h of incubation, cell lysates were prepared by using radioimmunoprecipitation assay (RIPA)/PBS buffer (1% NP-40, 0.5% sodium deoxycholate, 0.1% sodium dodecyl sulfate [SDS]). Cell lysates (0.5 ml with total protein concentration of 2 mg/ml) were pre-cleared by adding 15 µl of Protein A/Protein G-Plus agarose beads (Santa Cruz Biotechnology) and incubated for 15 min at 4°C. After centrifugation, the pre-cleared lysates were transferred to a fresh tube with 20 µl of Chk1 or Chk2 antibody and rotated for 1 h at 4°C. Next, 50 µl of protein A/protein G-Plus agarose beads were added and rotated for 90 min at 4°C. The agarose beads were washed with RIPA/PBS buffer, and a K-LISA Checkpoint Activity Kit was used according to the manufacturer's instructions. Activity was determined by reading the absorbance at dual wavelengths of 450 and 570 nm with use of a conventional ELISA plate reader.

### Generation of stable clones of lung cancer cell lines with short interfering RNA-mediated Chk2 gene silencing

To generate cell lines with stable knockdown of Chk2 expression, we initially screened four short interfering RNAs (siRNAs) cloned into double (U6-H1) promoter expression vectors purchased from System Biosciences (Mountain View, CA). Briefly, the vector was digested with BbsI and ligated with the annealed Chk2 siRNA designed based on web-based tools. All sense oligos had AAAG at the 5′ end, and all antisense oligos had AAAA sequence at the 5′ end. We also included a mutant as control. The sequences were as follows: CHK2Si1:5′aaagGAACCTGAGGACCAAG3′; CHK2ASi1:5′aaaaCTTGGTCC TCAGGTTC3′; CHK2Si2:5′aaagCGCCGTCCTTTGAATA 3′; CHK2ASi2:5′aaaaTATTC AAAGGACGGCG3′; CHK2Si3:5′aaagAACGGTATTATACAC CHK2ASi3:5′aaaaGTG TATAATACCGTT 3′; CHK2Si4:5′aaagAGTTTCAAGATCTTCTGTC 3′; CHK2ASi4:5′a aaaGACAGAAGATCTTGAAACT 3′; CHK2Simta:5′aaagGCTAGCTAGTCGATC 3′; CHK2Simtb:5′aaaaGATCGACTAGCTAGC3′. Stable clones were selected with use of puromycin (1 µg/ml); and after they were established, they were screened for expression levels of Chk2 protein relative to control. The clone with the best silencing effect (CHK2Si1 and CHK2ASi1) was chosen for the functional studies.

#### Transient silencing of NPRL2 gene expression in lung cancer cell lines

For transient knockdown of NPRL2 expression, we used synthetic double stranded siRNAs to target the NPRL2 coding mRNA sequence and the corresponding scrambled siRNAs were used as non-specific controls. A SMART-pool of four On-target NPRL2-specific siRNAs were used for this study: the target sequences were 5′GCAUCGAACACAAGAAGUA 3′, 5′GCAAGACAGGCAUGAGCUA 3′, 5′GUACUACGGCGUUGUGACA 3′, and 5′GAC CCAAGAUCACCUAUCA 3′. These siRNAs were purchased from Dharmacon (Lafayette, CO). H1299 cells were co-transfected with NPRL2 expressing plasmid and NPRL2-siRNAs, and empty plasmid vector and scrambled siRNAs were used as controls, respectively. Cells were first transfected with NPRL2 plasmid vector using FUGENE 6 (Roche, Indianapolis, IN) with 2.5 µg of DNA for 2×10^5^ cells per well in a 6-well plate. Six hours after plasmid transfection, the cell culture medium was replaced with fresh one without antibiotics and cells were then transfected with siRNAs using DharmaFECT transfection reagent with 50 µM of SiRNA complexed with 5 µl of DharmaFECTper well in the above 6-well plate. Cells were collected 24, 48, and 72 hours after transfection for preparation of crude protein lysates for Western blot analysis.

### Immunoprecipitation-Western blot analyses

H1299 cells were treated with 4 µg of empty vector or NPRL2 with or without 3.0 µM cisplatin. After 72 h of incubation, cell lysates were prepared by using RIPA/PBS buffer (1% NP-40, 0.5% sodium deoxycholate, 0.1% SDS). After blocking with 1 µg of normal IgG and 20 µl of protein A/protein G-Plus agarose beads (Santa Cruz Biotechnology), protein was immunoprecipitated from 500 µg extracts by using 2 µg of anti-Cdc2 or anti-cyclin B1 antibody (Santa Cruz Biotechnology) and 30 µl of protein A/protein G-Plus agarose beads for 5 h at 4°C. The agarose was collected by centrifugation and then washed four times with ice-cold PBS buffer. Next, the immunoprecipitates were resuspended in 1× SDS sample buffer and heated with 50 mM DTT at 95°C for 5 min; Western blotting was performed as described previously [20].

### Analysis of cell cycle kinetics

Changes in cell cycle kinetics in tumor cells treated with NPRL2 with or without cisplatin were analyzed by flow cytometry (with use of a fluorescence-activated cell sorter [FACS]) using an APO-BRDU KIT (BD Biosciences Pharmingen). Briefly, cells were plated in 60-mm dishes at 4×10^5^ cells per dish; the following day, the cells were treated with 4 µg of empty vector or NPRL2-expressing vector with or without cisplatin. At designated time points, cells were harvested and fixed in 1% paraformaldehyde. After incorporation of 5-bromo-2′-deoxyuridine 5′-triphosphates (BrdUTPs), cells were visualized with use of FITC-labeled anti-BrdU antibody. The amount of total cellular DNA was obtained by staining cells with PI/RNase buffer. Cells were processed for FACS analysis by terminal deoxynucleotidyltransferase-mediated deoxyuridine 5-triphosphate (dUTP) nick-end labeling (TUNEL staining) to determine cell cycle kinetics, as described previously [20].

### Animal studies

All animals were maintained and experiments performed according to National Institutes of Health and institutional guidelines established for the Animal Core Facility at The University of Texas M. D. Anderson Cancer Center. The animals used in this study were female *Nu/nu* mice (4–6 weeks of age) that were purchased from Charles River Laboratories (Wilmington, MA). Before tumor cell inoculation, mice were subjected to 3.5 Gy of total body irradiation from a cesium-137 radiation source to completely render the immune system of nude mice from rejection of tumor xenografting. To evaluate the efficacy of systemic administration of NPRL2 and cisplatin in an orthotopic model of pleural dissemination, the mice received an intrathoracic injection (27-gauge needle) of a suspension of H322 cells at a density of 2×10^6^ cells per 100 µl. The mice were randomly divided into the following 4 groups: A, treated with LacZ; B, treated with LacZ and cisplatin; C, treated with NPRL2; and D, treated with NPRL2 and cisplatin. Each treatment group consisted of 2 mice.

On day 26, we administered various nanoparticles (DOTAP:Chol-DNA complex) intravenously in tail veins of all mice at a dose of 25 µg of plasmid DNA (LacZ or NPRL2 plasmid) and 10 nmol of DOTAP:Chol each in 100 µl of 5% dextrose in water per mouse. In groups B and D, we injected cisplatin (2.5 mg/kg body weight) intraperitoneally along with the nanoparticles. To estimate γ-H2AX and phospho-Chk2 (Thr68) expression in tumors, the mice were sscrificed 48 h later, and the pleural tumors (larger than 5 mm) of each group were randomly harvested and flash frozen. γ-H2AX expression was estimated by anti-phospho-histone H2AX (Ser139) (1∶100) antibody and donkey anti-mouse IgG-FITC. The samples were then mounted with VECTASHIELD with DAPI to counterstain the nuclei. Immediately, the slides were examined with use of a fluorescence microscope. Also, phospho-Chk2 expression was analyzed by anti-phospho-Chk2 (Thr68) antibody (1∶100) by using a VECTASTAIN Elite ABC kit (Vector Laboratories) and was then microscopically examined.

### Statistical analyses

All experiments were repeated at least two times with duplicate or triplicate samples. ANOVA and Fisher's test were used to compare the values of the test and control samples. *P*<0.05 was considered statistically significant. StatView 5.0 software (Abacus Concepts, Inc., Berkeley, CA) was used for all statistical analyses.

## Results

### Induction of caspase activities in response to treatment with NPRL2 and cisplatin

**Figure 1:**
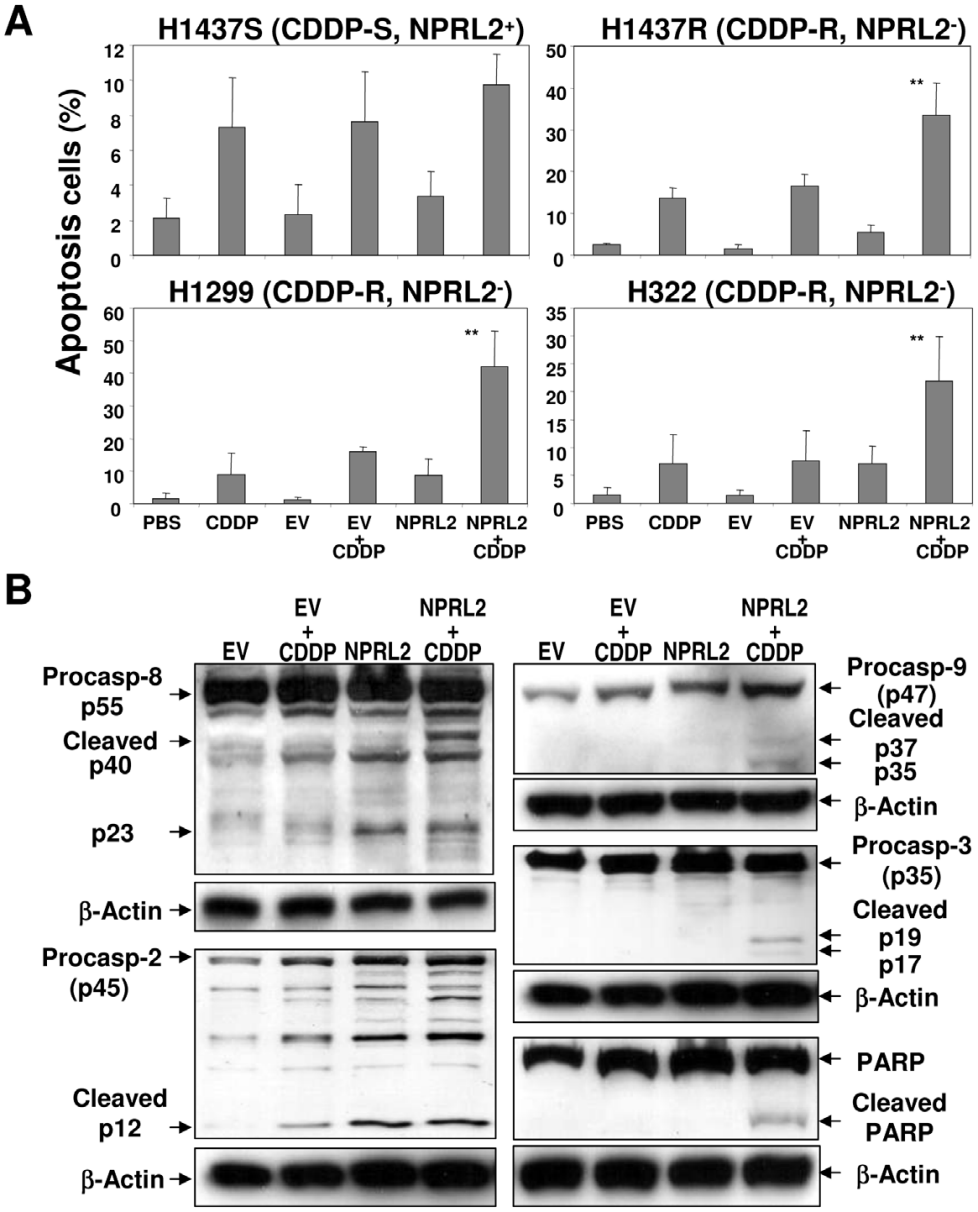
Induction of caspases by exogenous expression of NPRL2 and cisplatin in non-small cell lung cancer cells. (**A**) Induction of apoptosis in H1299 cells treated NPRL2 in the presence or absence of cisplatin (**CDDP**) by flow cytometry analysis with TUNEL staining. The PBS treated and empty vector (**EV**) treated cells were used as mock and negative controls, respectively. Error bars indicate SDs of the mean in three individual experiments (*, *P*<0.05; **, *P*<0.0005). (**B**), Activation of caspase cascades in H1299 cells by Western blot analysis. Several caspases in H1299 cells were examined by Western blotting 72 h after treatment with empty vector (**EV**) (with or without cisplatin) or with NPRL2 (with or without cisplatin). In cells treated with NPRL2 and cisplatin, caspases-3, -2, -8, and -9 and poly (ADP-ribose) polymerase (PARP) were cleaved. Also, caspase-2 was activated by treatment with only NPRL2.

NPRL2 sensitizes cisplatin-resistant cells (H1437R) to cisplatin-induced apoptosis (**Figure 1A**). Increased apoptosis was detected in all three NPRL2-negative, cisplatin-resistant cell lines treated with forced expression of NPRL2 and cisplatin. As shown in Figure 1A, apoptosis was induced in 33.4%, 42.0%, and 21.8% of H1437R, H1299, and H322 cells, respectively, after combined treatments. We also used Western blotting to determine the activation of caspases in H1299 cells treated with empty vector, empty vector plus cisplatin, NPRL2, or NPRL2 plus cisplatin (**Figure 1B**). Caspase-3, -8, and -9 were activated only in H1299 cells treated with NPRL2 and cisplatin, as shown by the cleavage products. Activation of caspase-2 was detected in cells treated with cisplatin or NPRL2 alone as well as the combination.

### Activation of ATM and NBS1 by NPRL2

**Figure 2:**
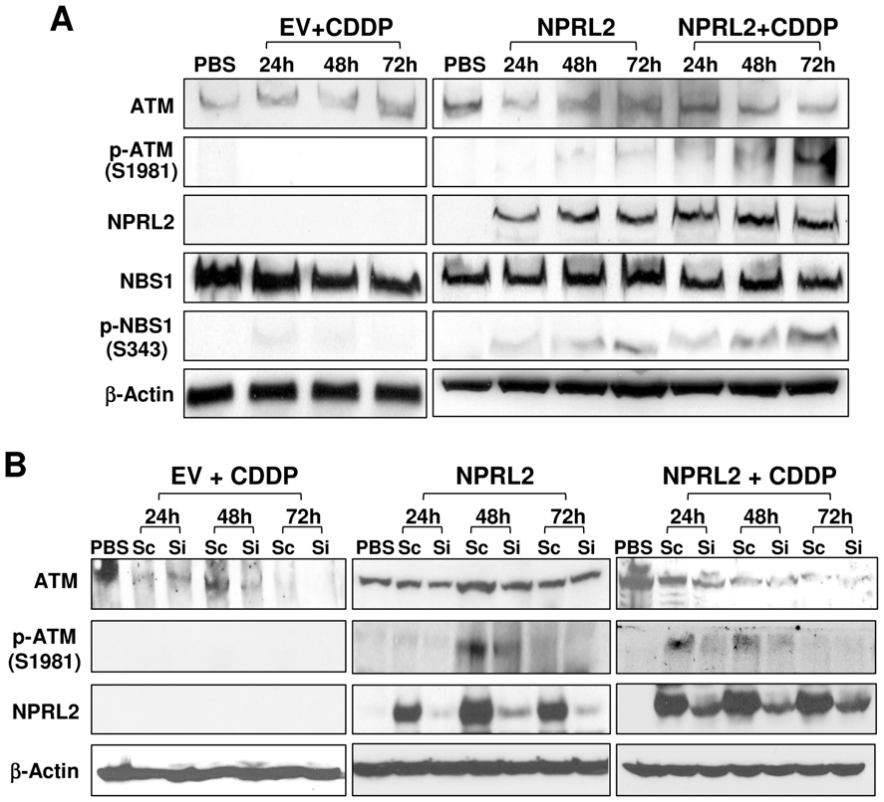
Activation of ATM signaling pathway by ectopic expression of NPRL2 protein. (**A**), Effects of ectopic expression of NPRL2 on expression and phosphorylation of ATM and NBS1 proteins.H1229 cells treated with an empty vector (**EV**) vector and IC_20_ dose of cisplatin (3.0 µM), NPRL2, or NPRL2 and IC_20_ dose of cisplatin were harvested at 24, 48, and 72 h after treatment and analyzed for expression of ataxia telangiectasia mutated (ATM) kinase, phospho-ATM (Ser1981), NBS1, and phospho-NBS1 (Ser343). β-actin was used as a loading control. Increases in phospho-ATM and phospho-NBS1 were observed in cells treated with NPRL2 or NPRL2 and cisplatin in a time-dependent manner, but not in cells treated with the control empty vector and cisplatin. (**B**), Effects of NPRL2-specific siRNA (**Si**) on expression of NPRL2 and ATM proteins in the presence and absence of cisplatin (**CDDP**). The scrambled siRNA (**Sc**) was used as a non-specific control.

We used Western blotting analysis to evaluate the activation of ATM in H1299 cells in response to ectopic activation of NPRL2 in the presence or absence of cisplatin (**Figure 2A**). In H1299 cells transfected with NPRL2, we detected a low level of phosphor-ATM (Ser1981) that had been activated by ATM kinase; after treatment with NPRL2 and cisplatin, the phosphor-ATM level markedly increased in a time-dependent manner. In H1299 cells treated with empty vector control and cisplatin, we detected no change in phosphor-ATM expression compared with the untreated control. We also examined the expression of phospho-NBS1 (Ser343) (Figure 2A), which is phosphorylated by activated ATM and recruited to sites of DNA damage to promote transmission of a series of DNA damage signals to downstream targets. Although phospho-NBS1 was not detected in H1299 cells treated with empty vector and cisplatin, it was detected in H1299 cells treated with NPRL2, and substantially increased in a time-dependent manner after treatment with NPRL2 and cisplatin.

The specificity of the NPRL2-mediated activation of the ATM signaling pathway was further confirmed by experiments using gene-specific siRNAs to knockdown NPRL2 expression in the presence and absence of cisplatin in these H1299 cells (**Figure 2B**). Ectopic activation of NPRL2 in NPRL2-deficient H1299 cells activated ATM, as indicated by the upregulated phospho-ATM expression at 24 h and 48 h post transfection in both the presence and absence of cisplatin. Knockdown of NPRL2 expression by NPRL2-specific siRNAs reduced or diminished the NPRL2-mediated upregulation of phospho-ATM expression, compared to those treated scrambled siRNAs controls at the same time points.

### NPRL2 enhances expression of γH2AX *in vitro* and *in vivo*

**Figure 3:**
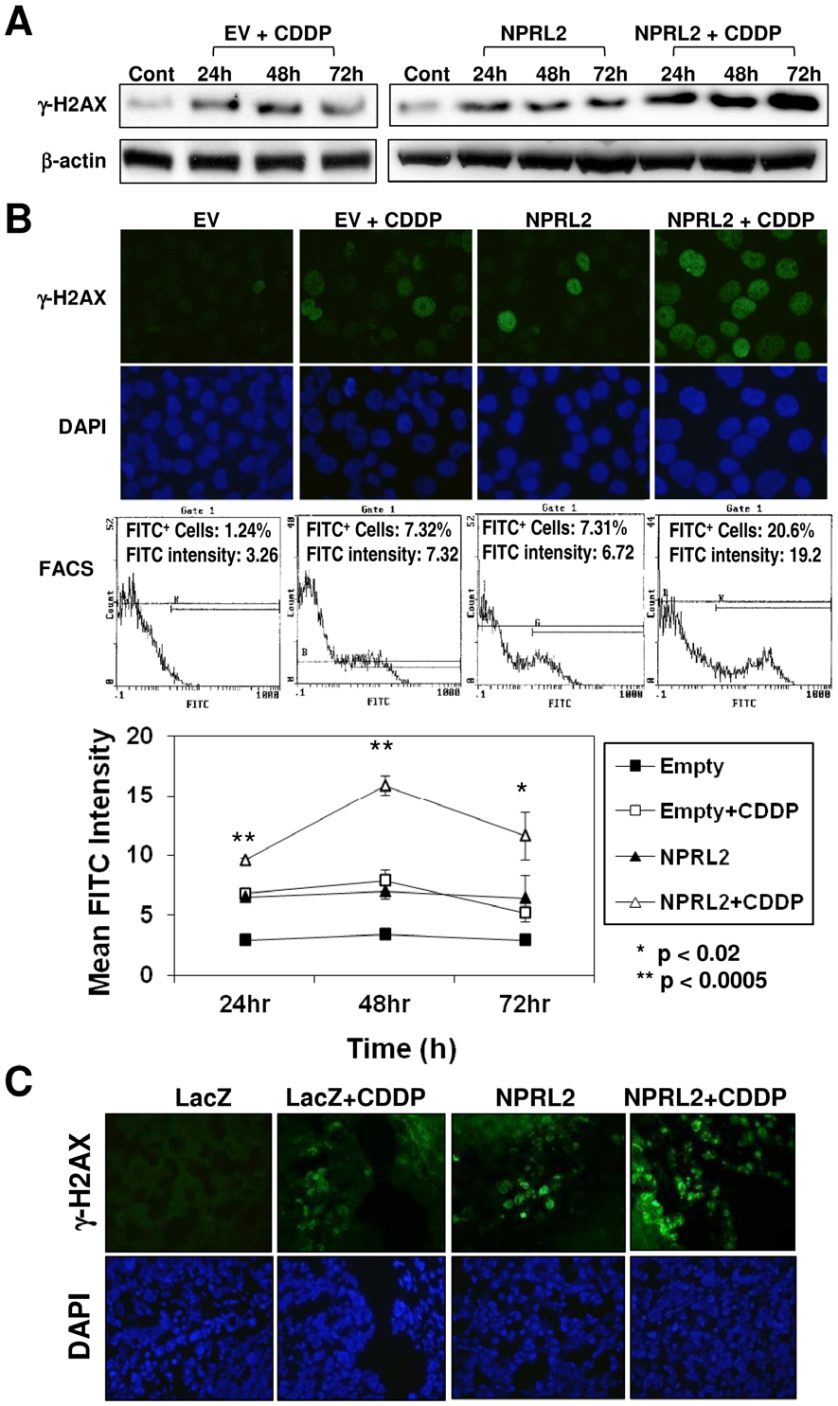
γ-H2AX, induced by cisplatin treatment, is also strongly enhanced by NPRL2. H1229 cells treated with an empty vector and IC_20_ dose of cisplatin (3.0 µM), NPRL2, or NPRL2 and IC_20_ dose of cisplatin were harvested at 24, 48, and 72 h after treatment. (**A**) Western blot shows remarkably enhanced expression of γ-H2AX in cells treated with NPRL2 and cisplatin compared with cells treated with empty vector and cisplatin. (**B**) γ-H2AX expression was examined at 48 h after treatment by immunofluorescence staining and flow cytometric analysis. γ-H2AX expression was detected in cells treated with empty vector and cisplatin or NPRL2 but not in cells treated with empty vector only, and this expression was dramatically enhanced in cells treated with NPRL2 and cisplatin. Magnification: ×800. Mean fluorescein isothiocyanate (FITC) intensity was also examined at 24, 48, and 72 h after treatment. At these three time points, γ-H2AX expression in cells treated with NPRL2 and cisplatin was significantly higher than that in cells in all other treatments (*P*<0.02 or *P*<0.0005). Bars, SDs of the mean in two individual experiments. (**C**) γ-H2AX expression was analyzed by immunofluorescence staining in an orthotopic model of H322 pleural dissemination, as described in the Materials and Methods section. The pleural tumor cells from the mice treated with LacZ and cisplatin or NPRL2 were weakly stained; in contrast, γ-H2AX was hyperexpressed in cells treated with NPRL2+ cisplatin. Magnification: ×400.

We used Western blotting for a time course evaluation of γ-H2AX protein expression (**Figure 3A**). In H1299 cells treated with empty vector and cisplatin or NPRL2, γ-H2AX protein levels increased and were the same by 24 and 48 h after treatment. However, by 72 h, the γ-H2AX protein levels in cells treated with empty vector and cisplatin decreased markedly, whereas levels in cells treated with NPRL2 remained almost the same as those at the 24-h and 48-h time-points. In cells treated with a combination of NPRL2 and cisplatin, γ-H2AX protein levels increased in a time-dependent manner.

Next, immunofluorescence staining and flow cytometric analysis for γ-H2AX expression at 48 h after treatment was performed (**Figure 3B**). Expression of γ-H2AX protein expression in the nuclei of cells treated with both NPRL2 and cisplatin was enhanced compared with that in all other groups. Furthermore, the mean FITC intensity of NPRL2 and cisplatin treatment was significantly elevated compared with that of all other treatments (*P*<0.02) during the time course.

γ-H2AX protein expression was then measured in a human NSCLC H322 orthotopic pleural tumor xenograft in mice. The H322 cell line is NPRL2-negative and cisplatin-resistant. On day 26 after intrathoracic injection of H322 cells, we administered NPRL2 or LacZ nanoparticles in the tail vein with or without intraperitoneal cisplatin injection. The pleural tumors from the mice treated with LacZ along with cisplatin or NPRL2 weakly stained for γ-H2AX (**Figure 3C**). Tumors treated with NPRL2 and cisplatin showed high levels of γ-H2AX (**Figure 3C**).

### Activation of Chk1 and Chk2 by NPRL2 and cisplatin *in vitro* and *in vivo*

**Figure 4:**
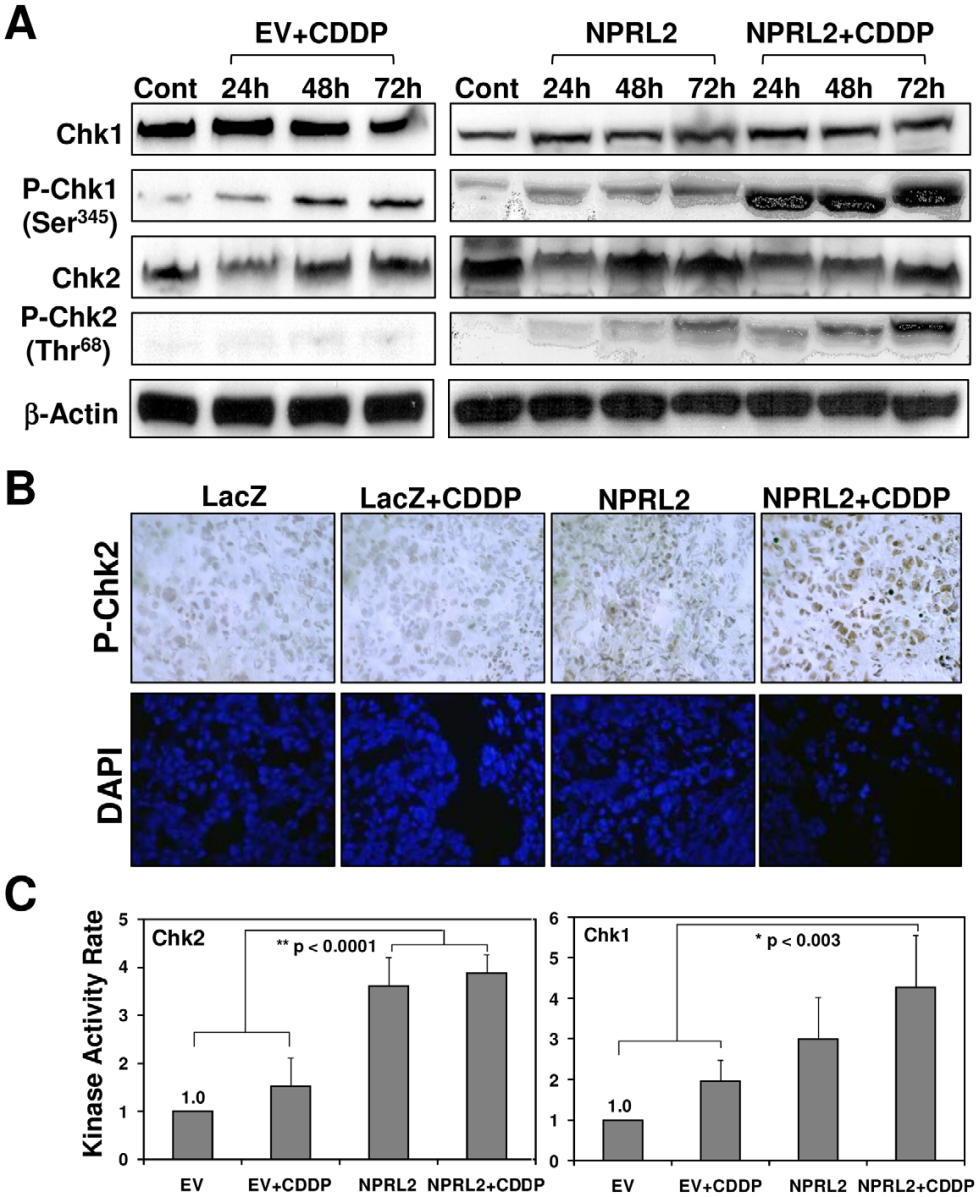
Combination of NPRL2 and cisplatin increases the activation of both Chk1 and Chk2. (**A**) H1229 cells treated with empty vector and IC_20_ of cisplatin (3.0 µM), NPRL2, or NPRL2+ with IC_20_ dose of cisplatin were harvested at 24, 48, and 72 h after treatment and analyzed for expression of Chk1, P-Chk1 (Ser345), Chk2, and P-Chk2 (Thr68). In cells treated with empty vector and IC_20_ dose of cisplatin or NPRL2, P-Chk1 increased in a time-dependent manner. In contrast, in those treated with NPRL2 and IC_20_ dose of cisplatin, P-Chk1 was dramatically enhanced. Although P-Chk2 was not detected in H1299 cells treated with empty vector and IC_20_ dose of cisplatin, it was clearly increased in a time-dependent manner in those treated with NPRL2 or NPRL2+ IC_20_ dose of cisplatin. (**B**) P-Chk2 expression was immunohistochemically analyzed in an orthotopic model of H322 pleural dissemination, as described in the Materials and Methods section. P-Chk2 expression was slightly detected in pleural tumor cells from mice treated with NPRL2 nanoparticles, but not in those treated with LacZ or LacZ + cisplatin. In contrast, treatment by NPRL2 nanoparticles and cisplatin induced high phospho-Chk2 expression in tumor cells. Magnification: ×400. (**C**) The kinase activity of Chk1 and Chk2 in H1299 cells treated with empty vector + IC_20_ dose of cisplatin, NPRL2 or NPRL2+ IC_20_ dose of cisplatin was analyzed with use of a K-LISA Checkpoint Activity Kit, an enzyme-linked immunosorbent assay (ELISA)-based activity assay. Chk1 kinase activity was greater in cells treated with NPRL2+ cisplatin than in cells treated with empty vector or empty vector + cisplatin (*P*<0.003). Chk2 kinase activity was more strongly enhanced in cells treated with NPRL2 or NPRL2+ cisplatin than in cells treated with empty vector or empty vector + cisplatin (*P*<0.0001). Bars, SDs of the mean in four individual experiments.

We examined the phosphorylation of Chk1 on Ser-345 and Chk2 on Thr-68 by Western blotting (**Figure 4A**). In H1299 cells treated with empty vector and cisplatin or NPRL2, phosphorylated Chk1 increased in a time-dependent manner. In cells treated with NPRL2 and cisplatin, Chk1 expression was greatly enhanced. Although phosphorylated Chk2 was not detected in H1299 cells treated with empty vector and cisplatin, it clearly increased in a time-dependent manner in cells treated with NPRL2 or NPRL2 with cisplatin. We evaluated phospho-Chk2 protein expression in the orthotopic tumor xenografts by immunohistochemical staining of tumor cells from each group (**Figure 4B**). The tumor cells from the mice treated with LacZ or LacZ and cisplatin had very low-level staining. For tumors treated with NPRL2, phospho-Chk2 expression was detected at a low level (**Figure 4B**). Treatment with combination NPRL2 and cisplatin induced high phospho-Chk2 expression.

We also measured the kinase activity of Chk2 and Chk1 in H1299 cells treated with NPRL2 with or without cisplatin with use of the K-LISA Checkpoint Activity Kit, an ELISA-based kinase activity assay. Chk2 kinase activity in cells treated with NPRL2 or NPRL2 and cisplatin was strongly enhanced compared with activity in cells treated with empty vector with or without cisplatin (*P*<0.0001) (**Figure 4C**). Chk1 kinase activity in cells treated with NPRL2 and cisplatin was stronger than in cells treated with empty vector with or without cisplatin (*P*<0.003) (Figure 4C).

**Figure 5:**
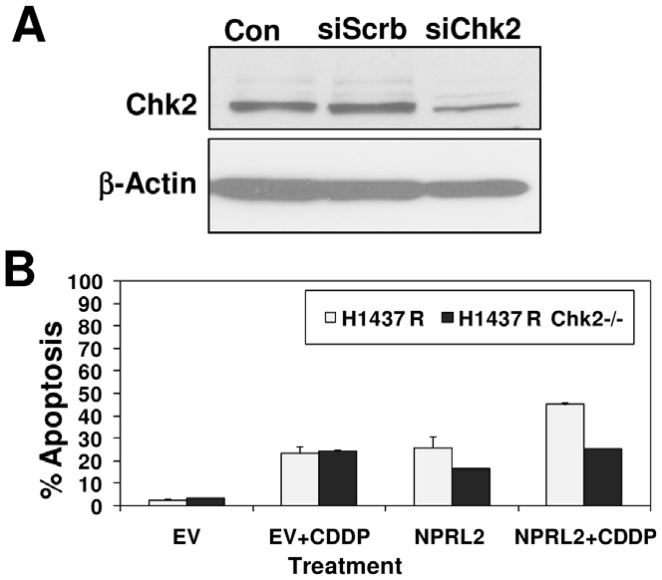
Lung cancer cell line H1437R was used to analyze apoptosis by flow cytometry after treatment with NPRL2 and IC_20_ dose of cisplatin. (**A**) Western blot shows reduction in Chk2 expression using SiRNA specific to Chk2 (SiChk2) and scrambled siRNA (SiScrb) as control in stable clones generated in 1437R lung cancer cell line. (**B**) Measurement of cellular apoptosis by fluorescence-activated cell sorter (FACS) analysis with TUNEL staining. Results show that down-regulated Chk2 expression attenuated NPRL-2-induced apoptosis and its ability to sensitize tumor cells' response to cisplatin.

To further define the nature of Chk2 interaction with NPRL2, we analyzed the effect of NPRL2 and Chk2 expression on apoptosis by using Chk2-specific siRNA (**Figure 5A**) to knock down Chk2 expression in cisplatin-resistant H1437 cells (H1437R) (**Figure 5B**). The level of apoptosis was determined by FACS analysis with terminal deoxynucleotidyltransferase-mediated deoxyuridine 5-triphosphate (dUTP) nick-end labeling (TUNEL) staining. The down-regulated Chk2 expression attenuated NPRL-2-induced apoptosis and its ability to sensitize the tumor cells' response to cisplatin.

### Enhanced effect of cisplatin in activating cell cycle checkpoints and fostering G2 arrest by NPRL2

**Figure 6:**
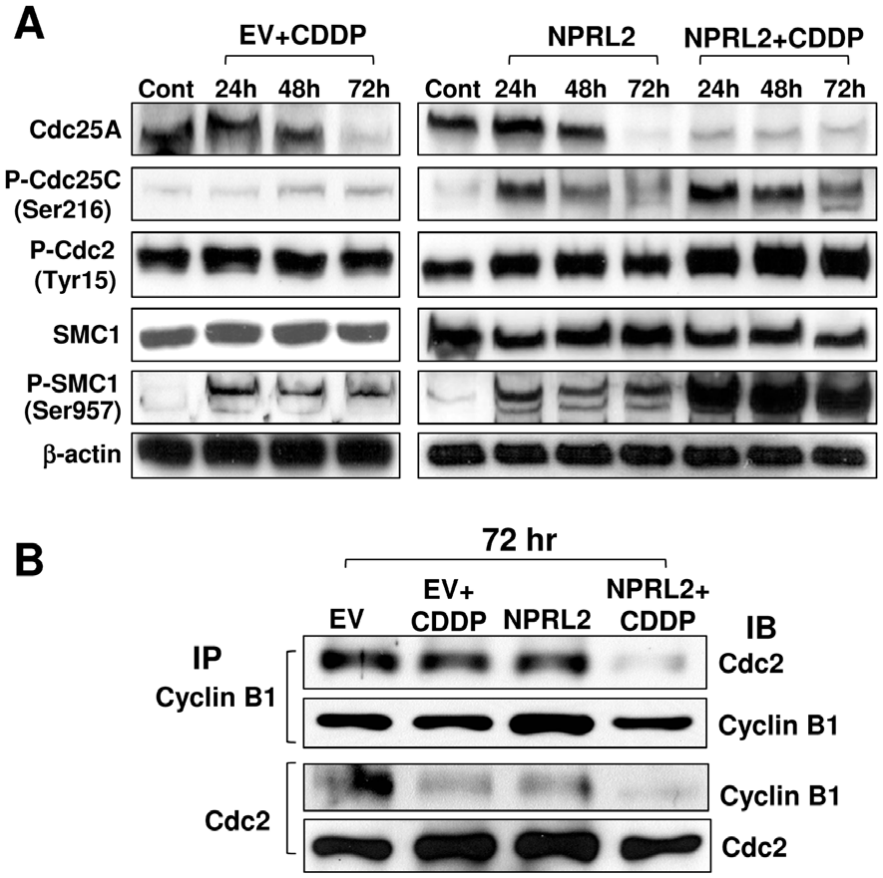
NPRL2 enhances the effect of cisplatin that can activate cell cycle checkpoints. H1229 cells treated with empty vector + IC_20_ dose of cisplatin (3.0 µM), NPRL2, or NPRL2+ IC_20_ dose of cisplatin were harvested at 24, 48, and 72 h after treatment and analyzed by Western blotting for expression of cell cycle signaling molecules. Control H1299 cells were treated with empty vector and harvested at 72 h after treatment. β-actin was used as a loading control. (**A**) Cdc25A was degraded by the treatment of NPRL2 or IC_20_ dose of cisplatin 72 h later, and this degradation in treatment of NPRL2+ cisplatin strongly appeared 24 to 72 h later. P-Cdc25C was slightly increased by treatment with cisplatin; in contrast, it was remarkably increased by treatment with NPRL2 or NPRL2+ cisplatin. P-Cdc2 and P-SMC1 were clearly enhanced more with NPRL2+ cisplatin treatment than with cisplatin or NPRL2 treatment. (**B**) Immunoprecipitation Western blotting (IP-WB) analysis for protein-protein interaction between Cdc2-cyclin B1. H1299 cells were transfected with either empty vector or NPRL2 plasmid with or without IC_20_ value of cisplatin. The backbone plasmid vector without NPRL2 was used as a transfection control. Protein extracts were collected 72 h after transfection and immunoprecipitated with either anti-Cdc2 or anti-cyclin B1 antibody and immunoblotted with Cdc2 or cyclin B1 antibody. NPRL2 and cisplatin treatment remarkably degraded the interaction of Cdc2/cyclin B1 complex.

To study the effect of NPRL2 on cell cycle kinetics, we examined the expression of Cdc25A, phospho-Cdc25C (Ser-216), phospho-Cdc2 (Tyr-15), and phospho-SMC1 (Ser-957) by Western blotting (**Figure 6A**). Cdc25A expression was reduced after treatment with NPRL2 or cisplatin at 72 h after treatment, but this reduction occurred at 24 h with combination treatment. Although phospho-Cdc25C expression was slightly increased by cisplatin, it was markedly increased after treatment with NPRL2 or NPRL2 and cisplatin. Moreover, expression of both phospho-Cdc2 and phospho-SMC1 increased more after treatment with NPRL2 and cisplatin than after treatment with either cisplatin or NPRL2. We examined whether NPRL2 influences the interaction of the Cdc2/cyclin B1 complex by using immunoprecipitation (**Figure 6B**). NPRL2 and cisplatin treatment reduced the binding of Cdc2 and cyclin B1 more effectively than did all other treatments.

**Figure 7:**
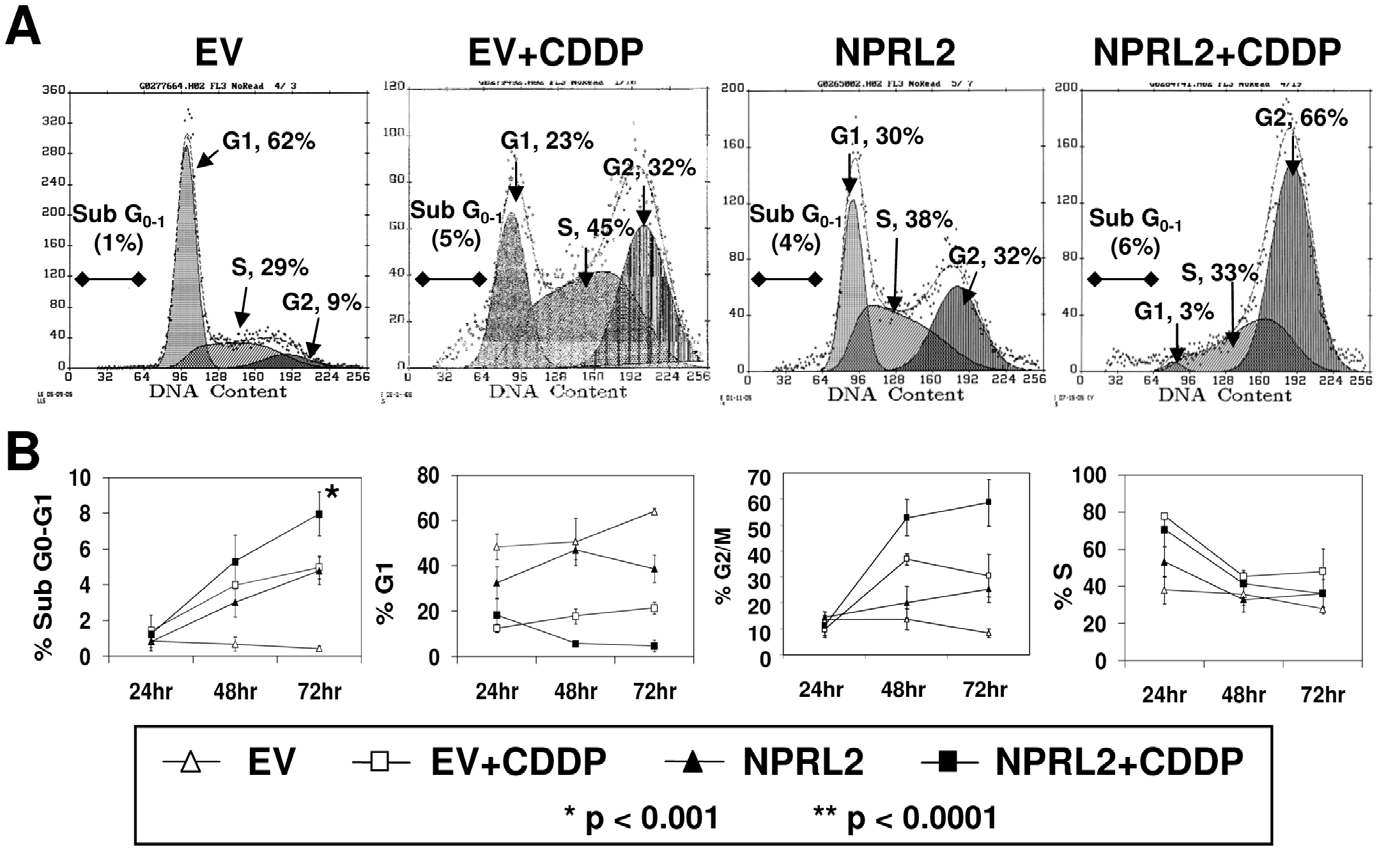
Combination treatment of NPRL2 and cisplatin induces strong cell arrest in G2/M phase. The cell cycle in tumor cells treated with NPRL2 and IC_20_ value of cisplatin was analyzed by flow cytometry with use of the APO-BRDU KIT. (**A**) The shift in cell cycle parameters of cells treated with NPRL2 is manifested as clear G1 and G2 peaks 72 h after treatment. In contrast, the cell cycle distribution showed an increase in the G2/M population after treatment with empty vector and cisplatin compared with cells treated with NPRL2 alone. The shift in cell cycle distribution was seen in cells treated with NPRL2+ cisplatin: the G2/M phase population strongly increased, whereas the G1 phase population decreased. (**B**) The cell cycle shift was analyzed at 24, 48, and 72 h after treatment. The combination of NPRL2 and cisplatin significantly increased the G2/M population in a time-dependent manner (*P*<0.001 at 48 h; *P*<0.0001 at 72 h) and increased the sub-G0-G1 population (*P*<0.001 at 72 h), compared with all other treatments. Bars, SDs of the mean from three individual experiments.

Next, the cell cycle in tumor cells treated with NPRL2 and cisplatin was analyzed by flow cytometry (FACS) with use of a APO-BRDU KIT (**Figure 7A**). The shift in cell cycle kinetics of cells treated with NPRL2 was manifested as clear G1 and G2 peaks at 72 h after treatment, consistent with the cells accumulating at the G1-S and G2-M checkpoints in response to inactivation of Cdc25A and Cdc25C by NPRL2 (**Figure 7A**). In contrast, the cell cycle distribution showed an increase in the G2/M population after treatment with cisplatin, compared with cells treated with NPRL2 alone. The combination of NPRL2 and cisplatin, however, increased the G2/M population more than either NPRL2 or cisplatin alone did, and in a time-dependent manner (*P*<0.001) (**Figure 7B**). In addition, the combination treatment increased the sub-G0-G1 population more than all other treatments did (*P*<0.001), and in a time-dependent manner (**Figure 7B**).

## Discussion

In this study, we showed that exogenous NPRL2 expression in NPRL2-negative tumor cells activated the DNA damage pathway, as evidenced by activation of ATM and NBS1 and increased γ-H2AX expression in lung cancer cells after NPRL2 treatment. NPRL2 treatment resulted in accumulation of cells at the G1-S and G2-M checkpoints associated with inactivation of Cdc25A and Cdc25C and degradation of the cyclin-Cdk complex. Treatment with only NPRL2 induced strong activation and cleavage of caspase-2, suggesting that the activation of caspase-2 may be one of the early NPRL2-mediated molecular events. Caspase-2 is required to translocate Bax protein to the mitochondria, permeabilize the mitochondrial membrane, and release apoptogenic factors such as cytochrome C from these organelles [21], [22]. These results indicate that the essential function of exogenous NPRL2 involves the activation of the DNA damage checkpoint pathway, which regulates not only cell cycle checkpoints but also DNA repair, genome maintenance, senescence, and apoptosis [17].

One potentially important means by which DNA adducts caused by cisplatin may kill cells is by induction of programmed cell death or apoptosis [23]. It is generally accepted that the main mechanisms affecting the occurrence of cisplatin resistance include increased drug efflux, decreased drug influx, increased cellular glutathione and metallothionein levels, increased DNA repair, and oncogene expression [24]. However, emerging evidence suggests that a significant number of cases of cisplatin resistance might be the result of a defective apoptotic program. In such cases, increased levels of DNA damage would be required to induce signal-initiating apoptosis [25]. Indeed, we demonstrated that after cisplatin treatment, phosphorylated ATM and NBS1 were expressed at very low levels.

Phosphorylation of histone H2AX by activated ATM, referred to as γ-H2AX, is dispensable for the initial recognition of DNA damage and recruitment of the mediators of the DNA damage checkpoint, including specific DNA-repair complexes, to the site of DNA damage [10], [12], [13], [26]. Cells from mice that lack the *H2AX* gene manifest a G2/M checkpoint defect similar to that in ATM-deficient cells exposed to low doses of ionizing radiation (12). γ-H2AX appears during apoptosis concurrently with the initial appearance of high-molecular-weight DNA fragments. Therefore, γ-H2AX formation is an early chromatin modification event that follows initiation of DNA fragmentation during apoptosis before DNA repair [27].

Adding exogenous NPRL2 to cisplatin treatment induces three key events in the DNA damage pathway. First, this combination increases γ-H2AX expression compared with cisplatin-only treatment. It has been reported that γ-H2AX formation is induced by apoptotic DNA fragmentation, a critical event that occurs during apoptosis. Electroporation of DNase I or restriction enzymes into cells is sufficient to induce the formation of γ-H2AX, suggesting that mere activation of diverse apoptotic nucleases should generally result in γ-H2AX formation [27]. Furthermore, it was reported that γ-H2AX formation is a cellular response to endonuclease-mediated DNA fragmentation downstream of caspases in the course of apoptosis [27]. Gamma-H2AX hyperphosphorylation is strongly correlated to apoptotic chromatin fragmentation, and this phosphorylation in apoptotic cells depends indirectly on activation of caspases and nuclear scaffold protein [28]. We found that the effector caspase, caspase-3, is activated and PARP is cleaved only in NPRL2 and cisplatin combination treatment. Also, PARP was cleaved in cells treated with exogenous NPRL2 and cisplatin, suggesting that treatment of NPRL2 and cisplatin enhances γ-H2AX expression and induces strong apoptosis.

Second, adding NPRL2 treatment to cisplatin significantly enhances Chk1 and Chk2 kinase activity. We were able to detect phospho-Chk2 expression in metastatic tumors in an orthotopic model, even though it was not detected after treatments with only cisplatin.

Third, the combination of NPRL2 and cisplatin increased the G2/M population in a time-dependent manner compared with treatment of cisplatin only.

The activation of Chk1 and Chk2 after DNA damage is dependent on the activated ATM or ATR protein kinase [29]. Chk2 is phosphorylated on Thr-68, which thereby promotes the oligomerization of Chk2 by creating a specific binding site for the FHA domain of another Chk2 molecule. The homodimerization of Chk2 also facilitates transphosphorylation of the C-terminal kinase domain (Thr-383, Thr-387), which is required for full activation of Chk2 [30], [31]. In contrast, Chk1 is activated by phosphorylation on Ser-317 and Ser-345 [32]. Activated Chk1 and Chk2 can regulate cell cycle arrest in late G1, intra S, and G2 phases through phosphorylation of substrates such as Cdc25A and Cdc25C [33], [34]–[38]. Furthermore, activated Chk2 can regulate not only p53-mediated apoptosis, but also p53-independent apoptosis [38].

The family members of Cdc25 such as Cdc25A and Cdc25C promote cell cycle progression by activation of the cyclin-dependent kinase Cdk2 and Cdc2 (Cdk1) through dephosphorylation of inhibitory phosphorylation at Thr14 and Tyr15 [33], [39], [40]. Degradation of Cdc25A, resulting from phosphorylation by the activated Chk2 and/or Chk1, leads to sustained inhibitory phosphorylation of Cdk2 and Cdk1 and cannot activate the Cdk2/cyclin E or Cdc2/cyclin B complex, leading to apparent p53-independent late G1 arrest [41], an S-phase delay [42], or G2 arrest [36]. Phosphorylation of Ser-216 on Cdc25C by activated Chk1 and/or Chk2 creates a binding site for 14-3-3 protein, and this interaction is believed to result in persistent cytoplasmic Cdc25C localization, thus preventing the G2/M transition since the Cdc2/cyclin B complex cannot be activated [33], [43], [44]. SMC1 is phosphorylated on Ser-957 and Ser-966 by activated ATM in an NBS1-dependent manner, and this phosphorylation of SMC1 is required for activation of the intra-S checkpoint [16]. The intra-S checkpoint is regulated by two parallel pathways mediated by ATM-NBS1-SMC-1 and ATM/ATR-Chk1/Chk2-Cdc25A [42]. Combination treatment of NPRL2 and cisplatin strongly inactivates Cdc25A and Cdc25C. Also, phospho-Cdc2 (Tyr-15), which is an inactivated type of Cdc2, is clearly increased with NPRL2 and cisplatin treatment but not with cisplatin treatment only.

Moreover, we found that with immunoprecipitation of Cdc2 and cyclin B1, combination NPRL2 and cisplatin treatments degraded the interaction of Cdc2 and cyclin B1, leading to inactivation of the Cdc2/cyclin B1 complex and arrest in G2/M. Proliferating cells treated with cisplatin arrest in G2/M [45], [46]. Cisplatin-induced apoptosis is dependent on the presence of cells in the S and G2/M phases of the cell cycle, even in a cell type that seemed “primed” for the induction of apoptosis [47]. Also, we previously reported that lung cancer cells arrested at the G2/M phase ultimately underwent apoptosis with characteristic nuclear fragmentation [48].

Therefore, we hypothesize that combining NPRL2 with cisplatin can enhance the induction of apoptosis that essentially prevents the potentially dangerous passage of damaged cells into mitosis. Indeed, apoptotic cells increased 2- to 3-fold in cisplatin-resistant and NPRL2-negative cells transfected with NPRL2 compared with those treated with cisplatin alone. Moreover, tumor cells treated with NPRL2 and cisplatin have a remarkably high number of TUNEL-positive cells compared with those treated with cisplatin alone in an orthotopic model of cisplatin-resistant pleural dissemination (5).

**Figure 8:**
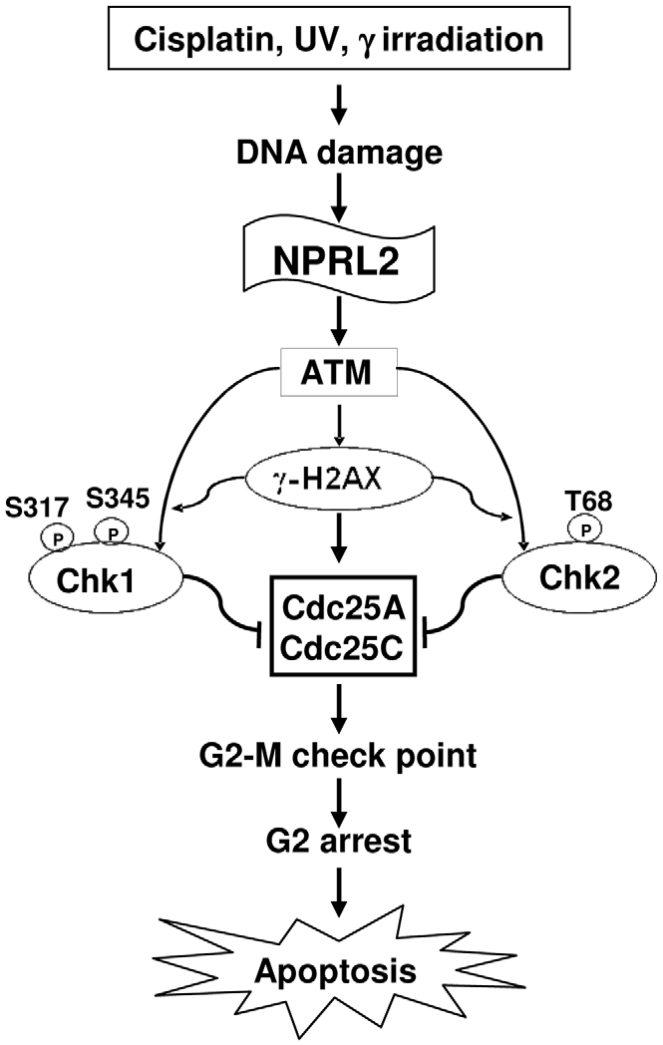
Prospective role of NPRL2 in combating resistance to cisplatin. NPRL2+ cisplatin (*cis*-diamminedichloroplatinum (II) [CDDP]) treatment effector caspase, caspase-3, is activated and PARP is cleaved. Therefore, our result that the combination treatment of NPRL2 and CDDP activates a caspase cascade and hyperphosphorylates H2AX suggests that this combination treatment can strongly enhance the apoptotic pathway. Adding NPRL2 treatment to CDDP significantly enhanced Chk2 and Chk1 kinase activity, and NPRL2+ CDDP treatment remarkably degraded the interaction of Cdc2 and cyclin B1, leading to the inactivation of the Cdc2/cyclin B1 complex and arrest in G2/M. The cells arrested at the G2/M phase ultimately promote apoptosis with characteristic nuclear fragmentation. This combination treatment strongly inactivates Cdc25A and Cdc25C, and phospho-Cdc2 (Tyr-15), an inactivated type of Cdc2, is clearly increased in NPRL2+ CDDP treatment.

The cell cycle in tumor cells treated with NPRL2 and cisplatin was analyzed by flow cytometry (FACS). The shift in cell cycle kinetics in cells treated with NPRL2 was manifested as clear G1 and G2 peaks at 72 h after treatment, consistent with the cells accumulating at the G1-S and G2-M checkpoints in response to the inactivation of Cdc25A and Cdc25C by NPRL2. In contrast, the cell cycle distribution showed an increase in the G2/M population after treatment with cisplatin, compared with cells treated with NPRL2 alone. It was reported that cisplatin-treated cells arrested in G2 in an attempt to repair cisplatin-induced DNA damage before passage into mitosis [49]. If cisplatin-induced cellular damage were irreparable during the G2 phase, controlled elimination of platinated cells *via* apoptosis would prevent the passage of these cells into mitosis [23], [50]. Therefore, it is interesting that the combination of NPRL2 and cisplatin increases the G2/M population in a time-dependent manner, suggesting that NPRL2 can enhance the induction of apoptosis, a potentially important process by which cisplatin-DNA adducts kill cells. In addition, this combination treatment increased the sub-G0-G1 population more than all other treatments did, and in a time-dependent manner.In summary, we hypothesize that NPRL2 activates the DNA damage checkpoint pathway in cisplatin-resistant and NPRL2-negative cells. The combination of NPRL2 and cisplatin can enhance and resensitize the response of cisplatin-nonresponders to cisplatin treatment through the strong activation of the DNA damage checkpoint pathway, leading to cell cycle arrest in the G2/M phase and induction of apoptosis (**Figure 8**) and suggesting that NPRL2 gene delivery for cisplatin-resistant patients may safely enhance the efficacy of cisplatin in the treatment of lung cancer.
